# Target Detection Using Ternary Classification During a Rapid Serial Visual Presentation Task Using Magnetoencephalography Data

**DOI:** 10.3389/fncom.2021.619508

**Published:** 2021-02-26

**Authors:** Chuncheng Zhang, Shuang Qiu, Shengpei Wang, Huiguang He

**Affiliations:** ^1^National Laboratory of Pattern Recognition and Research Center for Brain-Inspired Intelligence, Institute of Automation, Chinese Academy of Sciences, Beijing, China; ^2^School of Artificial Intelligence, University of Chinese Academy of Sciences, Beijing, China; ^3^Center for Excellence in Brain Science and Intelligence Technology, Chinese Academy of Sciences, Beijing, China

**Keywords:** RSVP, ERP, MEG, CNN, SVM

## Abstract

**Background:** The rapid serial visual presentation (RSVP) paradigm is a high-speed paradigm of brain–computer interface (BCI) applications. The target stimuli evoke event-related potential (ERP) activity of odd-ball effect, which can be used to detect the onsets of targets. Thus, the neural control can be produced by identifying the target stimulus. However, the ERPs in single trials vary in latency and length, which makes it difficult to accurately discriminate the targets against their neighbors, the near-non-targets. Thus, it reduces the efficiency of the BCI paradigm.

**Methods:** To overcome the difficulty of ERP detection against their neighbors, we proposed a simple but novel ternary classification method to train the classifiers. The new method not only distinguished the target against all other samples but also further separated the target, near-non-target, and other, far-non-target samples. To verify the efficiency of the new method, we performed the RSVP experiment. The natural scene pictures with or without pedestrians were used; the ones with pedestrians were used as targets. Magnetoencephalography (MEG) data of 10 subjects were acquired during presentation. The SVM and CNN in EEGNet architecture classifiers were used to detect the onsets of target.

**Results:** We obtained fairly high target detection scores using SVM and EEGNet classifiers based on MEG data. The proposed ternary classification method showed that the near-non-target samples can be discriminated from others, and the separation significantly increased the ERP detection scores in the EEGNet classifier. Moreover, the visualization of the new method suggested the different underling of SVM and EEGNet classifiers in ERP detection of the RSVP experiment.

**Conclusion:** In the RSVP experiment, the near-non-target samples contain separable ERP activity. The ERP detection scores can be increased using classifiers of the EEGNet model, by separating the non-target into near- and far-targets based on their delay against targets.

## Introduction

Rapid serial visual presentation (RSVP) is a high-speed brain–computer interface (BCI) experiment paradigm. In the rapid presented sequences, the odd-ball pictures can trigger the unique event-related potential (ERP) activity, known as P300 visual-evoked potentials in the brain (Won et al., 2019). This neural signal is generally chosen from a variety of well-studied non-invasive electroencephalography (EEG) and magnetoencephalography (MEG) signals (Lawhern et al., 2018). The detection of ERP onsets can be used to identify the pictures of interest in the sequence (Helfrich and Knight, 2019). As a result, the RSVP paradigm has been used in multiple BCI applications, e.g., picture identification, screen spellers, and other applications that require identifying target stimulus at high speed.

The applications of RSVP in BCI largely depend on the ERP detection accuracy. The machine learning methods have been widely used in ERP detection using the noisy single sample signals (Huang et al., 2011; Cecotti, 2016; Lin et al., 2017). Machine learning algorithm formulates the classifier to learn the ERP pattern in the high-dimensional neural signal, and automatically suppress the effect of noise. The xdawn algorithm was used to enhance ERP components in the EEG and MEG data. Support vector machine (SVM), linear discriminator (Cecotti, 2016), and convolution neural network (CNN) classifiers (Lawhern et al., 2018) have been applied to ERP detection tasks (Xiao et al., 2020). The weighted linear discriminant analysis has been used to reduce calibration time in the P300-based BCI paradigm; it not only reduces the computation request but also reduces the fatigue of subjects prior to BCI experiment (Jin et al., 2020b). Further, optimal feature selection method of common spatial pattern using L1-norm and Dempster–Shafer theory has been used in the EEG dataset to improve the robust against the non-stationary across time and subjects (Jin et al., 2020c). Despite the improvements in algorithm, it is still difficult to obtain the reliable ERP waveform from a single trial since the signal-to-noise rate is large in neural signal (Creel, 2019).

Besides the algorithm improvement, the paradigm of RSVP experiments also evolved. Jin et al. has developed a novel cheeks-stim paradigm for the P300 BCI experiment to substantially increase the efficiency and experience of BCI users (Lin et al., 2018; Jin et al., 2020a). Indeed, the reliable ERP can be obtained by averaging the waveform of several ERP trials, and there are RSVP paradigm improvements using the averaged multiple trials to increase the accuracy of ERP detection. Lin et al. developed a novel triple RSVP paradigm for the P300 BCI speller. It presented three single target character stimuli three times and uses the averaged signal to increase ERP detection accuracy (Lin et al., 2018). Cecotti et al. used the dual-RSVP paradigm. The sequence was presented synchronously with a fixed lag, and the succeeding two signals were used to increase the ERP detection accuracy (Cecotti, 2016). Additionally, the triple-RSVP paradigm has also been used to acquire higher accuracy (Mijani et al., 2019). It shows that the classifiers took the benefit from the dual sample combination and produced higher detection score. The new RSVP paradigm designs indeed improved the performance of the RSVP BCI application; however, it still left the difficulty of single sample ERP detection problem unsolved, which is important to common RSVP applications.

One of the main difficulties of ERP detection using a single trial is their complex dynamics (Barry and De Blasio, 2018), since they vary in latency and length across trials. The high-speed presented stimulus in the RSVP paradigm makes the stimulus closer with each other and the difference more ambiguous in temporal. Evenly, the presentation speed is becoming so fast that the ERP reaches its peak after the next stimuli onset, when the presentation rate is larger than 30 Hz. Thus, detecting the target samples against their neighbors is becoming more difficult and produces a higher error rate on the single-trial ERP detection.

In this study, we presented an RSVP experiment with MEG data acquired. The visual material is natural scene pictures with or without pedestrians, and the pictures with pedestrians were used as target pictures. We used a new training method to increase the ERP detection scores. In the new method, the samples were separated into three classes instead of two classes in the traditional method. They are target, near-non-target, and far-non-target samples. Thus, we used the classifier not only discriminating the target and other samples but also learning the difference between target samples and their neighbors. The SVM and CNN in EEGNet architecture classifiers were trained to detect ERP based on MEG data. The experiment results showed that the new training method improved ERP detection scores of the EEGNet classifier. The visualization results further explained the different underling of ERP detection of SVM and EEGNet classifiers.

## Materials and Methods

### Visual Stimuli and Procedure

The participants were seated in the MEG scanner, and a screen was in front at 680 *mm*. During the MEG scanning, they were required to gaze on the center of the screen. The rapid visual stimuli were presented on the screen using a rapid flashed sequence of pictures. The picture size was 500 × 500 *pixels*^2^ covering 150 × 150 *mm*^2^ areas in the screen; thus, it subtended the area of 12.6 × 12.6 *degrees*^2^ in visual angle. The flash rate of pictures was set as 10 *Hz*, and there were no gaps between two consecutive pictures.

All the pictures were selected from a dataset consisting of 1,400 colored street scene pictures. The pictures containing pedestrians were used as target pictures, and others were used as non-target pictures. There were 56 target pictures and 1,344 non-target pictures in the dataset.

**Figure 1:**
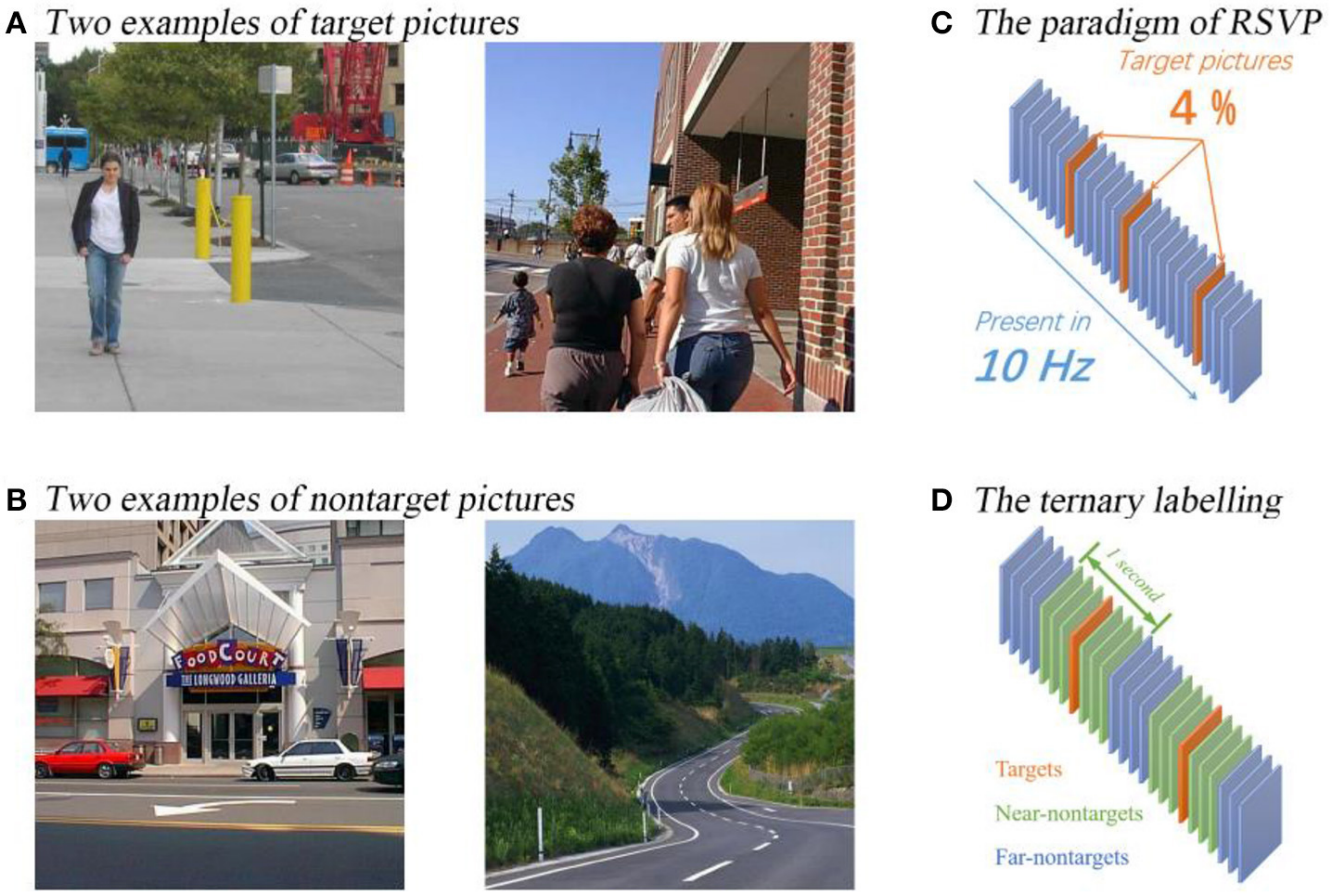
The paradigm and examples of pictures used in the RSVP experiment. The examples of target and non-target pictures are plotted on **(A,B)**. The paradigm is plotted on **(C)** and the ternary classification labeling protocol is plotted on **(D)**.

During a block, 100 pictures were shown in random order. The ratio of target pictures was set to 4%, resulting in 100 pictures with 4 target pictures and 96 non-target pictures. In every block, the 100 pictures were randomly sampled from the dataset without replacement. As a result, one session contained 14 blocks. During a session, the participants were required to press a button in their right hand when they were ready to start a block and press the same button when they see a target picture as soon as possible. The aim of asking participants to press the button is to keep them focused on the screen, and the button-pressing events were also recorded to make sure that the participant saw the target pictures instead of missing them. All the participants finished 11 consecutive sessions during the RSVP experiment. The paradigm of the RSVP experiment can be found in Figure 1.

### Participants

The experiment recruited 10 college students as participants in the RSVP experiment (seven males and three females, aged 23.79 ± 3.6) without previous training in the task. The participants practiced through a pseudo-RSVP block immediately before they entered the MEG scanner. The aim was only to make sure they had understood the rule of button pressing during the experiment. The participants exhibited normal or corrected-to-normal vision with no neurological problems and were financially compensated for their participation. The study was approved by the local ethics committee (Institute of Automation Chinese Academy of Sciences). All participants gave a written informed consent and received payment for their participation.

### MEG Acquirement and Preprocessing

During MEG experiment, subjects performed RSVP experiment in a MEG scanner. MEG recordings were conducted in a magnetically shielded room with a whole-head CTF MEG system with 272 channels (MISL-CTF DSQ-3500, Vancouver, BC, Canada) at the MEG Center of Institute of Biophysics, Chinese Academy of Sciences. Prior to data acquisition, three coils were attached to the left and right pre-auricular points and nasion of each participant, and a head localization procedure was performed before and after each acquisition to locate the participant's head relative to the coordinate system fixed to the MEG system. Participants were asked to lie in a supine position, and a projection screen was used to present visual stimuli during recording.

MEG data were recorded at a sampling rate of 1, 200 *Hz*, filtered between 0 and 600 *Hz*. We preprocessed the data using MNE software (Gramfort et al., 2014). The artificial noise of eye moving was suppressed using ICA method (Dimigen, 2020). Since ICA is sensitive to low-frequency drifts, the 1-Hz high-pass filter was used to suppress low-frequency signal prior to ICA fitting. Then, the sources with large skewness, kurtosis, and variance scores were marked and zeroed out from raw data. Then, the raw data were down-sampled to the sample rate of 100 *Hz*. The down-sampled data were then filtered by a band-pass filter to fetch data in the frequency band of 0.115*Hz*.

**Figure 2:**
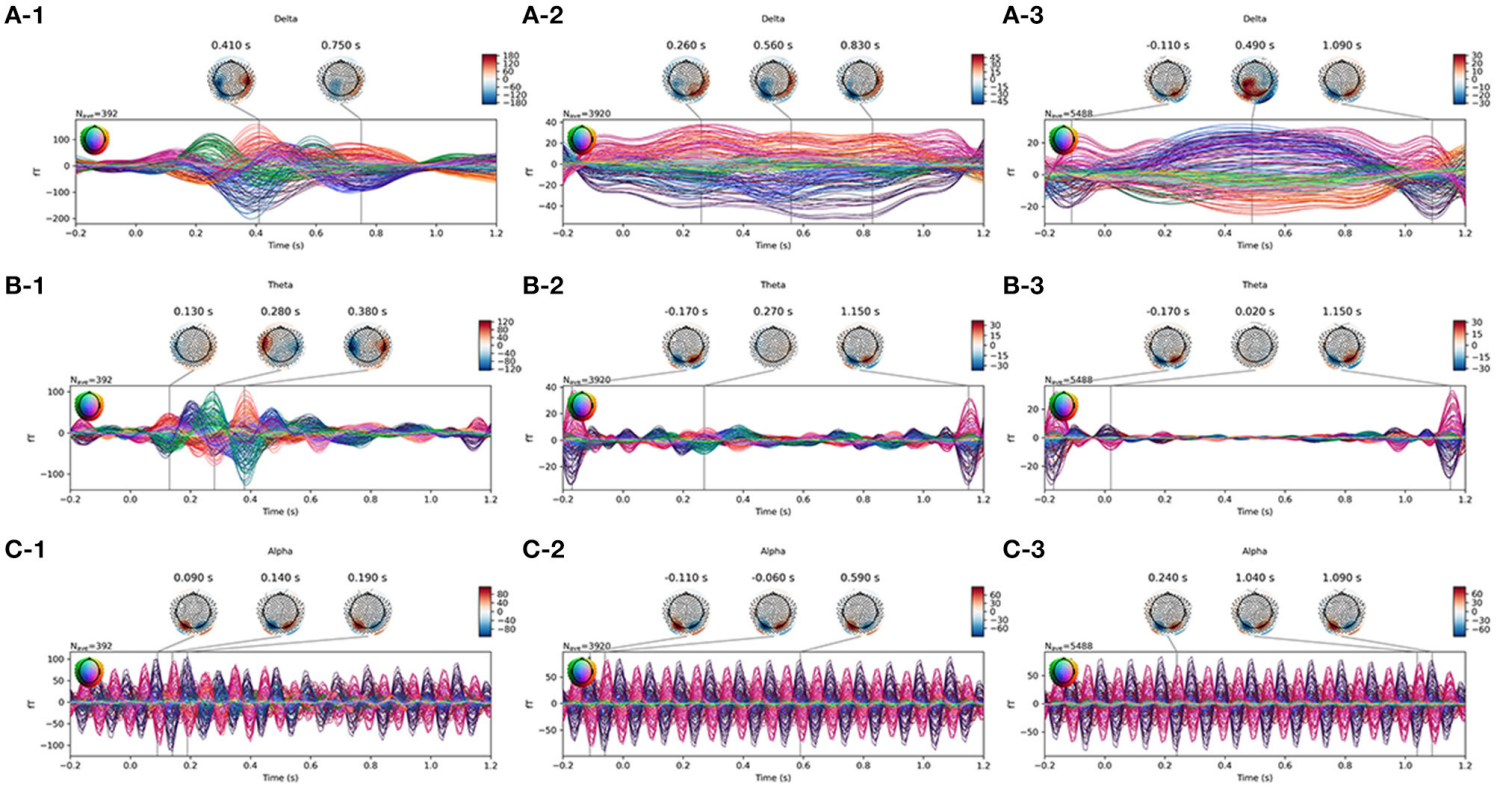
Average waveform visualization of MEG samples. The A/B/C row plots the average waveform of the frequency of Delta/Theta/Alpha band. The 1/2/3 column plots the average waveform of target/near-/far-non-target samples.

Data samples were then fetched from the filtered data. For every picture presented in the RSVP experiment, the time window ranging from −200 to 1, 200 *ms* from the onset was used to fetch the data sample. The samples also referred to the MEG epochs in some studies. The samples were baseline-corrected by the averaged value between −200 and 0 *ms* from the onset. The linear drifts were removed from the samples. As a result, the data sample could be represented by a matrix of 272 rows and 140 columns; 272 rows represented 272 channels and 140 columns represented 140 time points from −200 to 1,200 ms. The samples were then used to detect ERP activity. The averaged time series of the signals are plotted in Figure 2.

### ERP-Based Target Detection

#### MEG Sample Labeling

The lag between samples was 100 ms since the presentation rate was 10 Hz. However, the length of the samples was 1,400 ms. Thus, the samples inevitably overlapped with their neighbors. The traditional ERP detection method used dual classification, which only separated target and non-target samples, e.g., labeling target epochs as label 1 and non-target epochs as label 2. As a result, they used the same label to represent the non-target samples with or without ERP components. It forced the classifier to distinguish the ERP signals against their neighbors, which might contain the same ERP with a small latency. Thus, the confusion will inevitably decrease the accuracy of ERP detection.

In this study, we used three classification methods to further separate the target signal from their neighbors. Three labels were used in the experiment: target label (noted as 1), far-non-target label (noted as 2), and near-non-target label (noted as 3). The far-non-target samples refer to the epochs whose onset was far from target stimulus, which means that there were no target stimuli occurring within a 0.5-s range. The other non-target epochs were labeled as near-non-target labels. Simply, the target samples were ERP samples, the near-non-target samples contained ERP but of incorrect latency, and the far-non-target samples did not contain ERP activity.

#### ERP Detection Using SVM

The SVM is a widely used statistical learning algorithm, especially for large datasets with high dimensionality (Vapnik, 1998). It has been reported that SVM outperforms other competing methods in many researches (Williams, 2003; Pohlmeyer et al., 2011). The SVM has also been used for ERP detection in the RSVP experiment (Huang et al., 2011). Since the SVM was originally designed for binary classification, the trinary classification method used the one-against-one method that was proposed by Chih-Wei and Chih-Jen (2002) in the “libsvm” software package.

The prior feature extraction was also necessary for SVM classifier. We used signal enhancement with xdawn algorithm (Rivet et al., 2009). The xdawn method was used as a supervised feature extraction method to enhance the ERP components in the MEG data by maximizing the signal-to-signal-plus-noise rate (Cecotti, 2016). The number of components was set to six in this study based on prior research and visualization results. Thus, the 272-sensor MEG data were converted into six-component feature data to fit the SVM classifier.

SVM uses RBF kernel to explore more flexible classification strategy for high-dimensional data. In this study, we set the prior parameter gamma as “scale” to automatically calculate the variance of the training data. Since non-target samples were dozen times outnumbered target samples, we set the class-weight option as “balanced” to increase the weight of target signal in loss function to obtain a meaningful classifier.

#### ERP Detection Using EEGNet

EEGNet is an outstanding CNN architecture to detect ERP signal in EEG data (Lawhern et al., 2018). In this study, we used EEGNet to detect the ERP signal in MEG data. The EEGNet classifier was built and trained using “pytorch” toolbox in the high-performance GPU server. Since there were 272 sensors in the MEG data other than the 64 sensors in the EEG data, we changed the input number to 272 accordingly. Additionally, we used softmax function in the output to match the ternary classification. The loss function was calculated using the output of EEGNet and one hot-coded sample label. The architecture was the same as the “DeepConvNet” model of EEGNet (Lawhern et al., 2018). The parameters in the EEGNet were upgraded using the Adam optimizer. The learning rate was set as 0.001 for initiate and then the rate was set to shrink to 0.8 times every 50 epochs to avoid overfitting. The training process contained 500 epochs, and 300 training samples with equal class number were randomly selected in each epoch. Since the EEGNet classifier performed feature selection automatically using the first convolution layers, the band filtered MEG data were used directly without additional feature extraction process in prior.

#### Cross-Session Validation

We used the SVM and CNN model in EEGNet architecture classifier to detect ERP for identifying the target samples. To evaluate the reusability of the classifiers, we applied cross folder protocol to separate the MEG data into training and testing dataset recursively. The separation is based on the sessions of the experiment to keep the independency between the training and testing data. Since all the subjects finished 11 sessions of the RSVP experiment, we applied the 11-folder protocol. In each folder, the data of one session were used as testing dataset, and data of other sessions were concatenated to generate the training data.

In folders of 11 sessions, the following training and testing procedure were repeated. In the SVM part, the training dataset was used to train the xdawn spatial filter to perform feature extraction, and then the features were used to train an SVM classifier. The testing dataset was then applied by the trained xdawn spatial filter and SVM classifier to evaluate the detection scores. In the EEGNet part, the training dataset was used to train the parameter of the net without prior feature extraction and then the testing dataset was used to evaluate the detection scores.

As a result, we performed cross-session validation within subject to validate the discriminating power of the method. It was operated as the online experiment simulation. The model was fitted to samples in training sessions and then the test samples were transformed one by one to obtained the labels. Although the ternary classification gave labels of three class labels, we merged the near- and far-non-target labels as the non-target label. Thus, the ternary classification method was used to increase the discriminating power, and it was transparent to the experiment since it eventually produced binary labels.

Additionally, we also visualized the features to investigate the ERP detection underling of SVM and EEGNet classifiers. For the SVM classifier, the features extracted by the xdawn spatial filter were visualized. For EEGNet, the activity of the first convolution layer was visualized. We used the TSNE projection method to project the features into the two-dimensional manifold space. In the space, we showed the distribution of the target, near-, and far-non-target samples in a distance invariance manner.

## Results

### ERP Detection Scores

**Figure 3:**
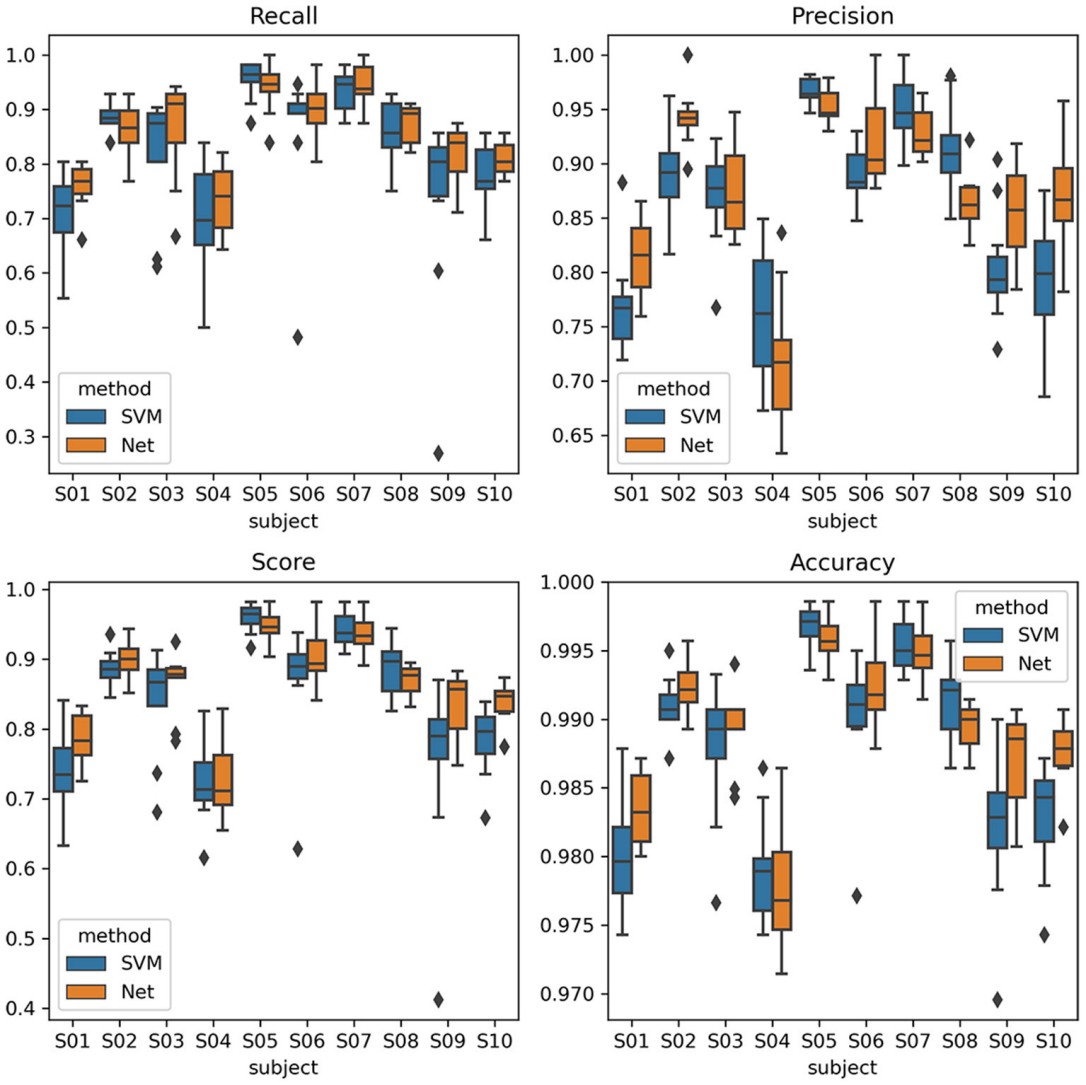
ERP detection scores with ternary classification of all the 10 subjects in box-and-whisker plots. The SVM labels refer to the score of SVM classifier, and the Net labels refer to the score of EEGNet classifier.

The ERP detection scores were recorded and compared between SVM and EEGNet classifiers. The scores of interests are the recall rate, precision rate, and F1 score of the target samples, which was also the aim of the RSVP experiment. The average scores of all the subjects were shown in Table 1. The recall score was higher for the EEGNet classifier. Additionally, the ternary classification method increased the scores of the EEGNet classifier beyond the SVM. The scores of EEGNet and SVM using ternary classification of all the subjects were plotted in Figure 3. It showed that the scores of EEGNet was higher than SVM on more subjects. The variance among cross-session folders of the EEGNet method were smaller. Moreover, the EEGNet with the ternary classification method also produced the highest F1 scores.

**Table 1:** Scoures table of ERP detection.

	**Recall**	**Precision**	**F1 score**	**Accuracy**
SVM (binary)	0.8206 ± 0.1304	0.8649 ± 0.0828	0.8364 ± 0.1027	0.9875 ± 0.0068
SVM (ternary)	0.8243 ± 0.1259	0.8610 ± 0.0823	0.8384 ± 0.1027	0.9876 ± 0.0070
Net (binary)	**0.8740 ± 0.0837**	0.7574 ± 0.1216	0.8085 ± 0.0987	0.9829 ± 0.0097
Net-3 (ternary)	0.8513 ± 0.0847	**0.8731 ± 0.0775**	**0.8608 ± 0.0749**	**0.9890 ± 0.0059**

*The bold values refer the highest value of the column*.

To make sure the comparison was valid, we applied analysis of variance (ANOVA) (Rouder et al., 2016) and paired *t*-test (Xu et al., 2017) method to test the statistical level of the difference between the scores. Firstly, to settle the complicity of the experiment, we used ANOVA to testify if the difference between the scores was because of the usage of classifiers. As a result, we used three-factor ANOVA; the factors were subject factor, folder factor, and method factor. The results showed that the method factor had main effect, which suggested that the choice of classifiers affected the scores. Then, we used the *t*-test method to obtain the *p*-value of the difference. The results showed that the increase of the EEGNet was significant since the *p*-value was < 0.001 for recall score and F1 score, please see Table 2 for the detail values.

**Table 2:** ANOVA tables of scores.

	***Df***	**sum_sq**	**mean_sq**	***F***	***PR* (>*F*)**
**Recall**					
Subject	9.0	1.1160	0.1240	21.4217	4.6338e−24
Method	1.0	0.0354	0.0354	6.1178	1.4347e−02
Folder	10.0	0.0956	0.0095	1.6530	9.5467e−02
Resibinary	173.0	1.0015	0.0057	NaN	NaN
**Precision**					
Subject	9.0	0.8201	0.0911	42.8921	1.3668e−39
Method	1.0	0.0071	0.0071	3.3651	6.8306e−02
Folder	10.0	0.0409	0.0040	1.9261	4.4532e−02
Resibinary	173.0	0.3675	0.002125	NaN	NaN
**F1 Score**					
Subject	9.0	0.9869	0.1096	37.6439	2.4480e−36
Method	1.0	0.0244	0.0244	8.3976	4.2433e−03
Folder	10.0	0.0620	0.0062	2.1310	2.4425e−02
Resibinary	173.0	0.5039	0.0029	NaN	NaN
**Accuracy**					
Subject	9.0	0.0058	0.0006	55.5035	1.9985e−46
Method	1.0	0.0000	0.0000	8.0118	5.1975e−03
Folder	10.0	0.0003	0.0000	2.7216	3.9466e−03
Resibinary	173.0	0.0020	0.0000	NaN	NaN

**Figure 4:**
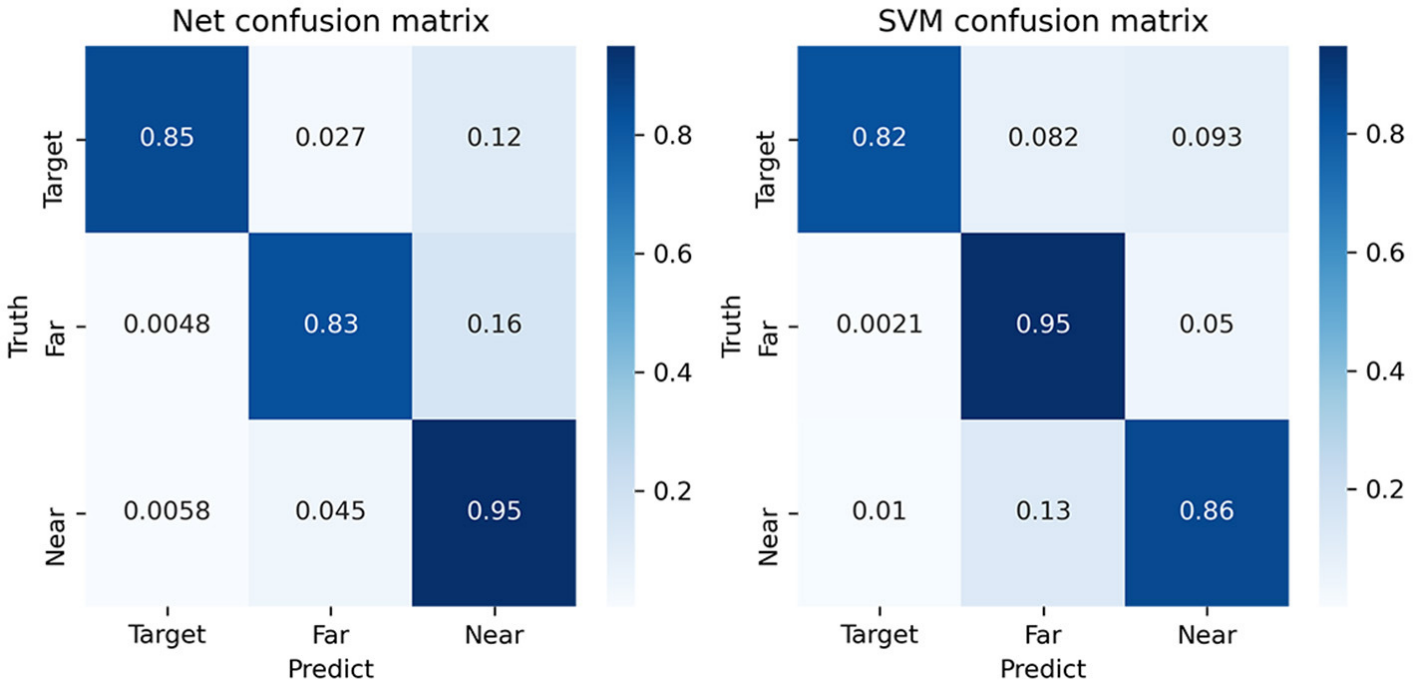
The average confusion matrix of the SVM and EEGNet method using ternary classification. The float numbers on the grids are the average value of percentage.

Figure 4 shows the confusion matrix of the classification. Firstly, it shows that the near- and far-non-target samples can be discriminated. The first row of the matrix had three columns, which showed the ratio of target samples being detected as target, near-non-target, and far-non-target samples. The second and third rows showed the ratio of near-non-target and far-non-target samples, respectively. As a result, the diagonal values were the ratio of the three classes of samples being correctly classified. The other values were the ratio of being incorrectly classified. The first row was used to calculate the scores of target samples classification. The value in the first column referred to the true-positive rate (TPR) (Albieri and Didelez, 2014). The value in the second and third columns referred to the false-negative rate (FNR) of target to near-non-target and far-non-target, respectively. The first column was used to calculate the scores of samples being classified to target samples. The false-positive rate (FPR) of near-non-target to target was the value of the second row and first column in the matrix.

The results showed that the TPR of target samples was higher in EEGNet, and the FNR of target to far-non-target was lower in EEGNet. According to the first column, the FPR of near-non-target to target is lower in EEGNet. According to the other elements in the matrixes, the discriminating power between target and near-non-target was also higher in EEGNet. It suggested that the higher scores of EEGNet were due to the fact that the new three classification method could increase the discriminating power between target and near-non-target of the EEGNet classifier.

**Figure 5:**
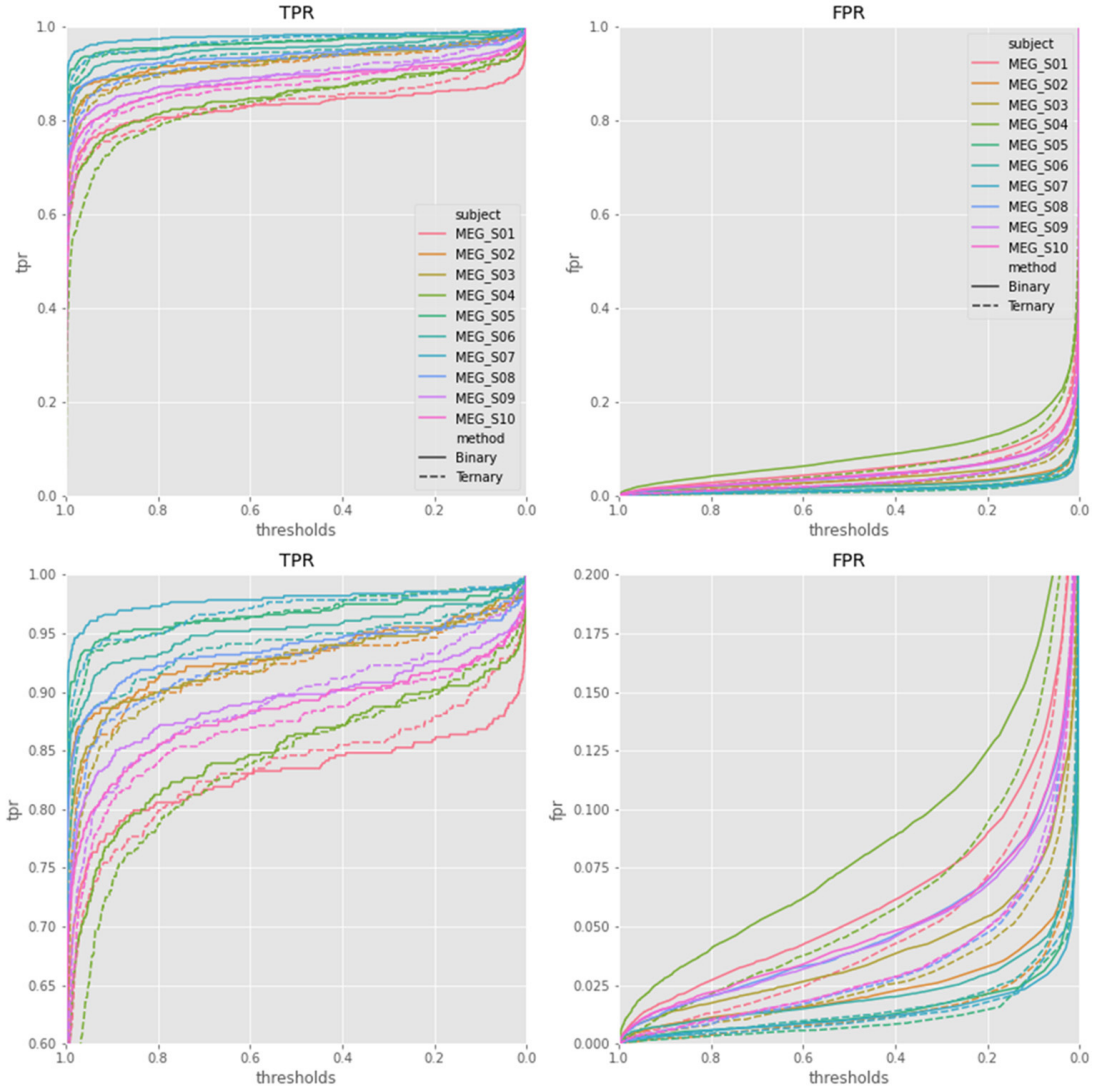
The TPR and FPR curves of EEGNet with thresholds of all the 10 subjects. The two plots on the bottom were the same as the plots on the top, other than using a smaller value range.

Additionally, since we used softmax function on the output layer of EEGNet, the probability of the sample as a target sample could be obtained. The TPR and FPR curves among different thresholds (Zhang et al., 2015) of target samples were plotted in Figure 5 based on the output of EEGNet. The area under curve (AUC) values of EEGNet were 0.9808 ± 0.0197 of binary classification and 0.9858 ± 0.0136 of ternary classification. The results showed that the ternary classification produced higher AUC values and lower FPR values than traditional binary classification protocol. The results suggested that the ternary classification method can largely suppress the FPR of target samples.

### Visualization

Figure 2 plots the waveform and topotactical activity of averaged samples of one subject on different frequency bands. The graphs used the joint plotting visualization method of MNE software, and the colors represented the 272 channels of the MEG set. The waveform of target samples on the Delta band clearly showed the ERP activity of the target pictures. The waveform on the Alpha band showed the SSVEP activity triggered by the 10-Hz presentation, and the SSVEP occurred in all the three kinds of samples. The differences between near- and far-non-target samples were mainly on the Delta band, and even their activities were both weak. It showed that the activity pattern of near-non-target samples was similar to target samples, and the far-non-target samples did not show similarity.

**Figure 6:**
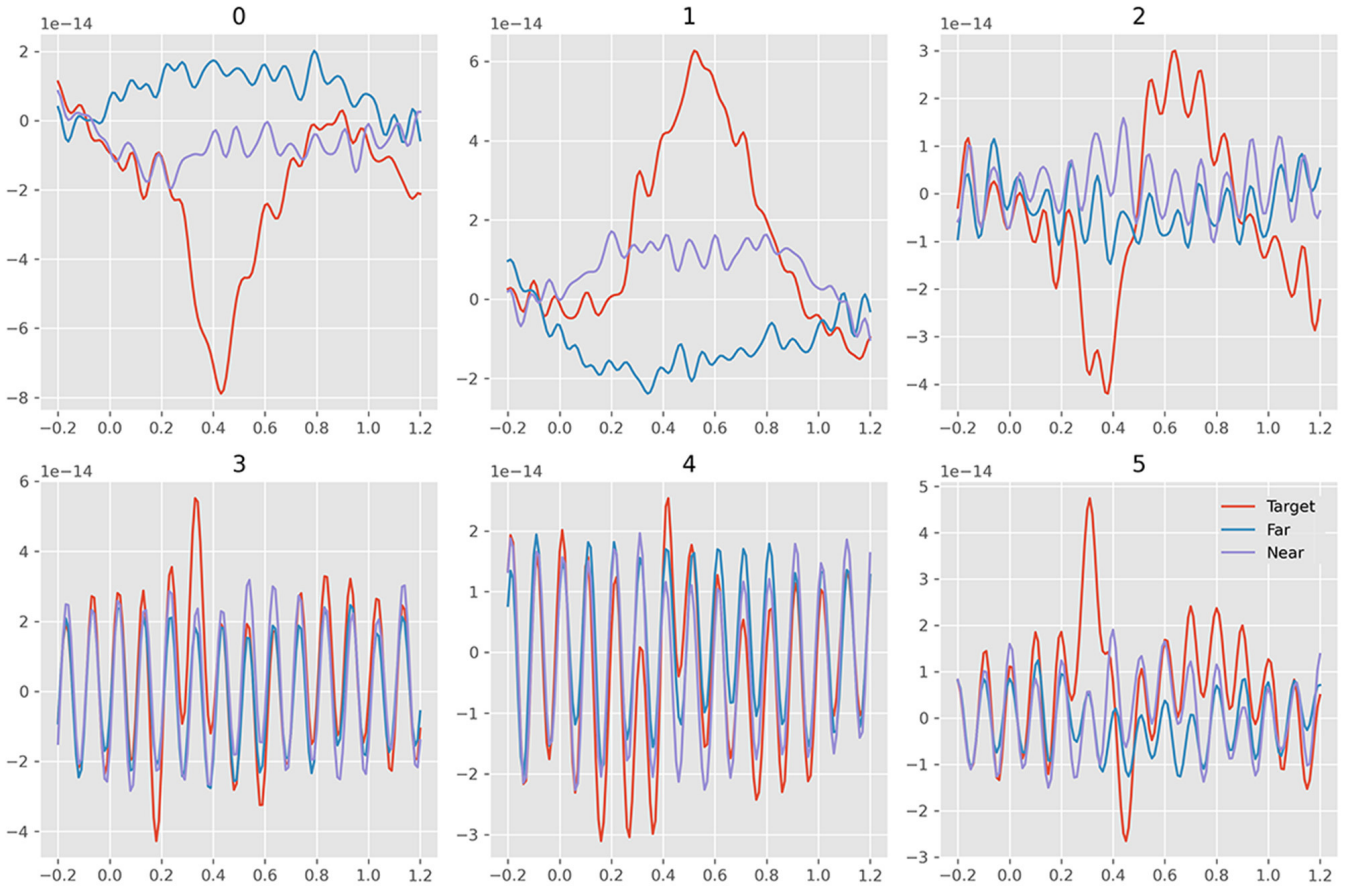
Average waveform plots of six xdawn features. The six grids refer to the six features; the colors refer to ternary kinds of samples.

**Figure 7:**
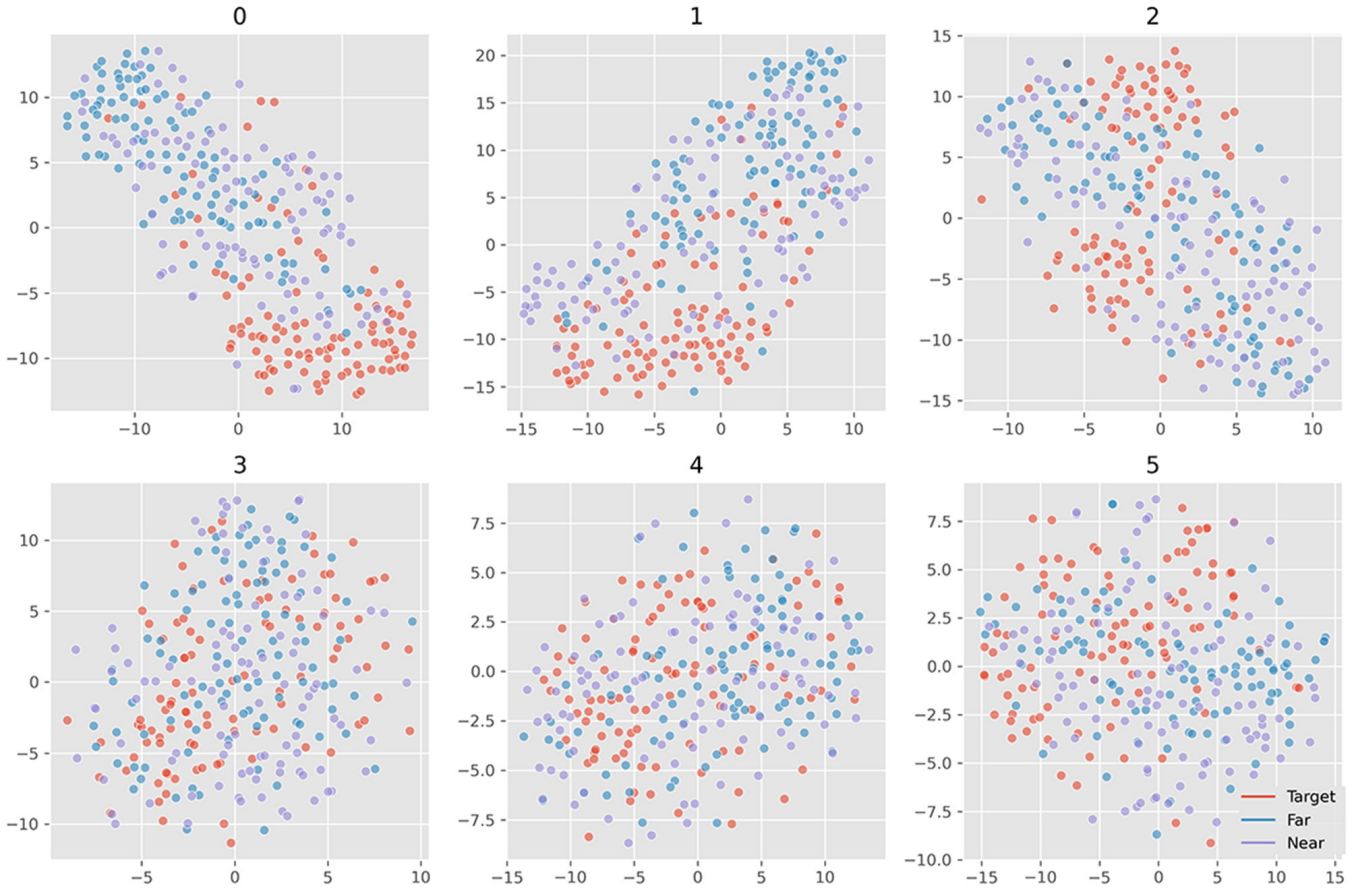
Projection of samples of six xdawn features in two-dimensional manifold space. The six grids refer to the six features; the colors refer to ternary kinds of samples.

Figure 6 plots all the six averaged components of xdawn extraction. The order was set as decreasing order of explained variance. It turned out that the first three features cover the main differences between target and non-target signals. There was little difference between near- and far-non-target samples. The SSVEP components mainly existed in the latter three features, which suggested that they were less important to ERP detection. Figure 7 plots samples in the two-dimensional manifold space. It showed a similar trend with the averaged plot. The first three features were more separated among the three kinds of samples.

**Figure 8:**
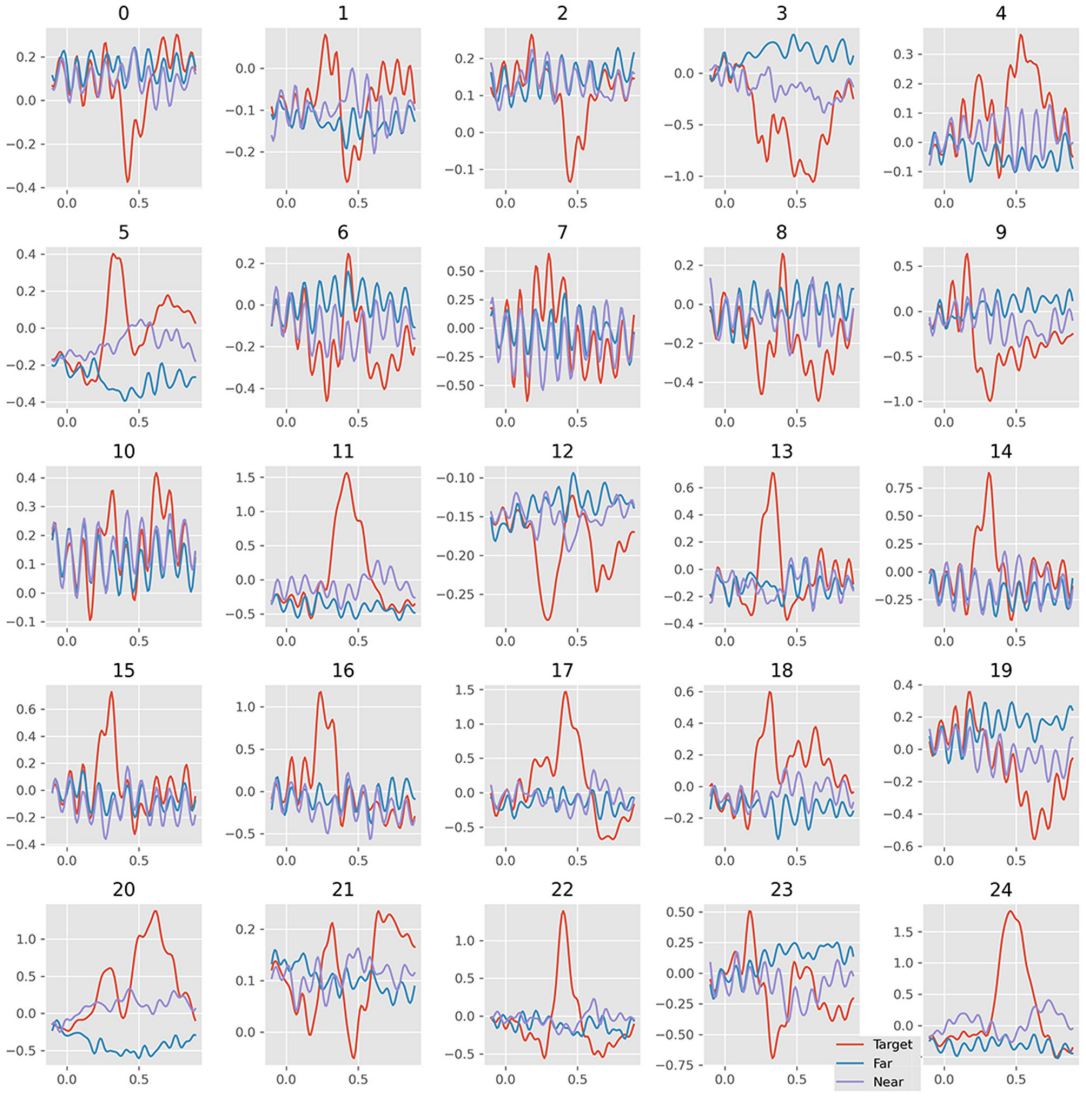
Average waveform plots of 25 EEGNet features. The 25 grids refer to the 25 features; the colors refer to ternary kinds of samples.

**Figure 9:**
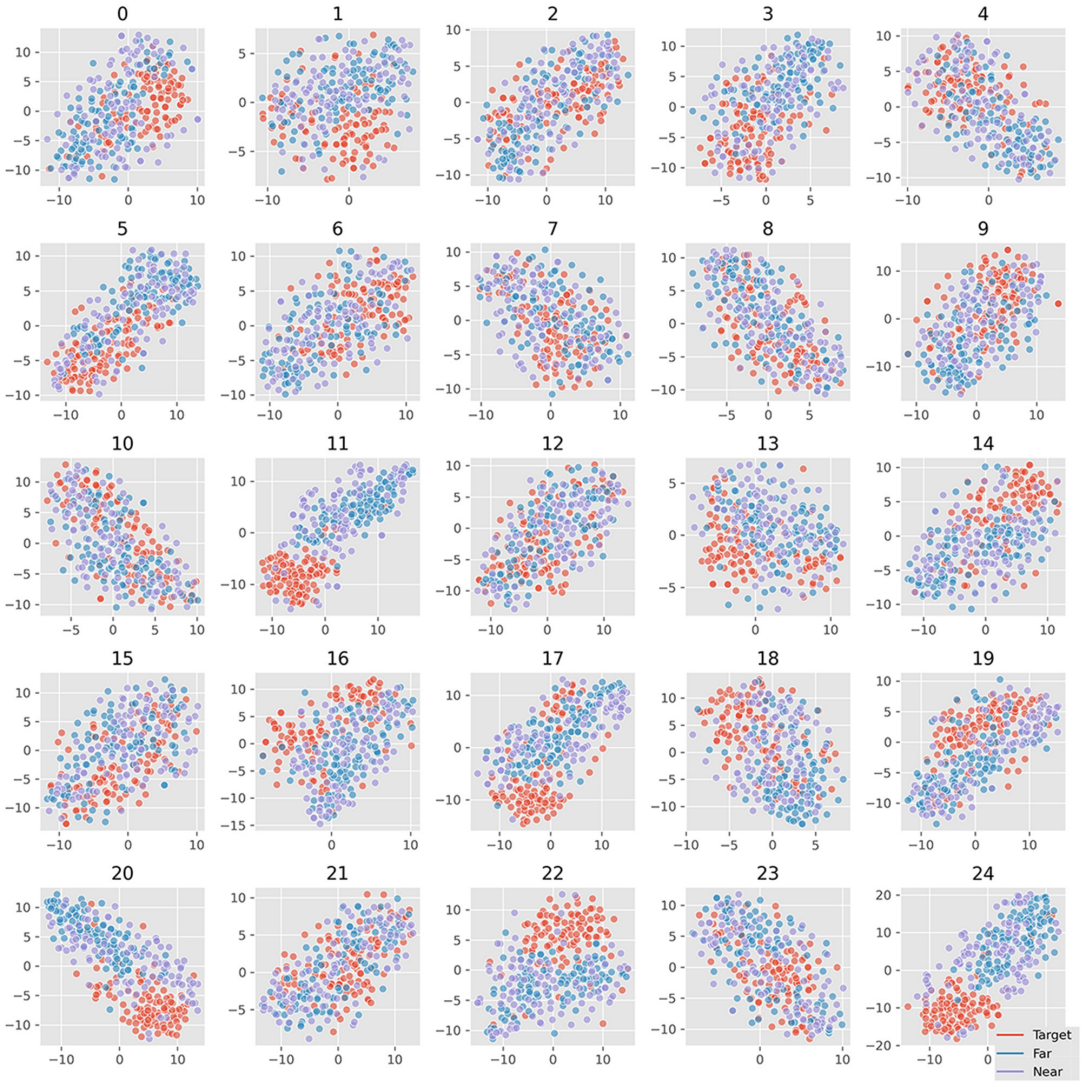
Projection of samples of 25 EEGNet features in two-dimensional manifold space. The 25 grids refer to the 25 features; the colors refer to ternary kinds of samples.

The visualization of EEGNet features was done in the same way as the SVM features. Figure 8 plots the waveform of the averaged 25 features. Figure 9 plots the features in the two-dimensional manifold space. It showed that all the 25 features show difference between three kinds of samples. The difference between near- and far-non-target samples was also clear. Moreover, the features containing SSVEP also showed difference among three classes. The features of No. 11, 13, 14, 15, 16, 17, 20, 22, and 24 showed a large difference between target and non-target samples. The features of No. 3, 5, 20, and 19 showed moderate difference between near- and far-non-target samples. The results were consistent with the confusion matrix of Figure 4, which showed large error rate between near- and far-non-target samples.

## Discussion

In this study, the MEG data were acquired during the RSVP experiment; the rapid presented pictures were natural scene pictures, and the pictures with pedestrians were used as target pictures. We presented the new ternary classification method to train the SVM and EEGNet classifiers to detect ERP signal to identify the onset of target stimulus. The new method has improved the detection scores using the EEGNet classifier.

The traditional machine learning method in the RSVP experiment only used binary classification to discriminate the target and other samples. The method ignores the similarity of target samples and their neighbors. The speed of RSVP in the experiment was 10 *Hz*. The latency between two samples was 0.1 s. However, the latency of a classic reliable ERP was about 0.3 s, which was widely known as the P300 feature signal (Mijani et al., 2019). The length of the ERP was not narrow either. As a result, the near-non-target samples inevitably contained the ERP the same as target samples (see the average waveform in Figure 2). The difference between them was only that the target samples contained the ERP with the “correct” latency, which was occasionally too small in some samples to distinguish them.

We separated the samples into three classes: target, near-, and far-non-target samples. The waveforms showed that the difference between them were mainly on the Delta band, and the near-non-target samples were more similar to the target samples (see Figure 2). The visualization of the features showed the difference between them either (see Figures 6–9). Thus, the non-target samples should be separated into two sets, the ones near a target sample (near-non-target) and others (far-non-target). The traditional methods did not separate the two kinds of non-target samples either. As a result, the classifiers had to solve the confusion by detecting some ERP signals and discarding others, which was bad to ERP detection.

The new ternary classification method trained the classifier to learn not only the difference between target and others but also the difference between target samples and their neighbors. It actually separated the ERP detection task into two folders. The first one was to detect ERP components in the samples to find target and near-non-target samples. The second one was to distinguish the two classes. The confusion matrix of EEGNet proved that the new method increased the TPR of target and near-target samples (see Figure 4).

Compared with the SVM classifier, the EEGNet provided higher TPR for ERP component detection. Although the TPR of far-non-target samples was lower than SVM, the incorrect samples were more likely to be classified as the near-non-target samples. Finally, the scores of target samples using EEGNet were overly higher than using the SVM classifier. The ROC plots of EEGNet showed the difference between the traditional binary and new ternary methods in detail (see Figure 5). The FPRs of target detection of the ternary method were largely lower than those of the binary method, while the TPRs of the two methods were similar. The results explained that the ternary method produced higher precision score than the binary method (see Table 1). It was shown that the TPR only reached 0.85 in confusion matrix (see Figure 5) and the overall accuracy reached 0.98 (see Table 1). The reason was the non-target samples largely outnumbered the target samples. Based on EEGNet classifier results, the TNR was extremely high (see the second and third row of the first column of the confusion matrix), which made the overall accuracy higher than the TRP value of target samples.

Based on the results of the study, the separation increased the ERP detection scores. The results suggested that the reason EEGNet produced higher ERP detection scores was that it had learned the difference between the samples with ERP and other samples without ERP signals. Furthermore, the results also suggested that the CNN model was better at detecting ERP components despite their variance in latency, which were consistent with the translation invariance of the CNN model (Furukawa, 2017). The visualization of 25 features of samples also verified that the CNN model can effectively extract the useful features automatically in the RSVP experiment (see Figure 9). As a result, the xdawn spatial filter was not necessary for the EEGNet classifier. Meanwhile, it also hinted that the CNN model could benefit from the correct separation of the samples.

The SVM classifier did not benefit from the ternary method. It might be due to the fact that SVM used time points in the samples as independent feature dimensions. The shifts of ERP components in near-non-target samples converted the feature from dimensions. Thus, it was hard for the SVM classifier to track the dependence between the time points. The reason we used xdawn in SVM classification was the lack of automatically extracting features of the SVM classifier (Bascil et al., 2016). The results also suggested that the six components had fully covered the explainable variance, and the increase of the components was not necessary.

## Conclusion

In this study, the MEG data were acquired during the RSVP experiment; the rapid presented pictures were natural scene pictures, and the pictures with pedestrians were used as target pictures. We also presented the new ternary classification method to train the SVM and EEGNet classifiers to detect ERP signal to identify the onset of target stimulus. We obtained a fair ERP detection accuracy using traditional SVM and EEGNet classifiers. The proposed ternary classification method showed the discrimination of the near- and far-non-targets in the RSVP experiment and increased accuracy in the EEGNet classifier. The visualization of the results also uncovered the different ERP detection underling between SVM and EEGNet classifiers.

## Data Availability Statement

The raw data supporting the conclusions of this article will be made available by the authors, without undue reservation.

## Ethics Statement

The studies involving human participants were reviewed and approved by Institute of Automation Chinese Academy of Sciences. The patients/participants provided their written informed consent to participate in this study.

## Author Contributions

CZ operated the experiment, analyzed the data, and wrote the manuscript. SQ operated the experiment. SW jointed in data analyzing. HH was in charge of the project. All authors contributed to the article and approved the submitted version.

## Conflict of Interest

The authors declare that the research was conducted in the absence of any commercial or financial relationships that could be construed as a potential conflict of interest.
